# Age differences in the effect of animacy on Mandarin sentence processing

**DOI:** 10.7717/peerj.6437

**Published:** 2019-02-14

**Authors:** Xinmiao Liu, Wenbin Wang, Haiyan Wang

**Affiliations:** 1 School of English for Specific Purposes, Beijing Foreign Studies University, Beijing, China; 2 National Research Centre for Foreign Language Education, Beijing Foreign Studies University, Beijing, China; 3 Language and Brain Science Center, School of Translation Studies, Qufu Normal University, Rizhao, Shandong, China

**Keywords:** Age, Animacy, Relative clause, Sentence processing

## Abstract

Animate nouns are preferred for grammatical subjects, whereas inanimate nouns are preferred for grammatical objects. Animacy provides important semantic cues for sentence comprehension. However, how individuals’ ability to use this animacy cue changes with advancing age is still not clear. The current study investigated whether older adults and younger adults were differentially sensitive to this semantic constraint in processing Mandarin relative clauses, using a self-paced reading paradigm. The sentences used in the study contained subject relative clauses or object relative clauses and had animate or inanimate subjects. The results indicate that the animacy manipulation affected the younger adults more than the older adults in online processing. Younger adults had longer reading times for all segments in subject relative clauses than in object relative clauses when the subjects were inanimate, whereas there was no significant difference in reading times between subject and object relative clauses when the subjects were animate. In the older group, animacy was not found to influence the processing difficulty of subject relative clauses and object relative clauses. Compared with younger adults, older adults were less sensitive to animacy constraints in relative clause processing. The findings indicate that the use of animacy cues became less efficient in the ageing population. The results can be explained by the capacity constrained comprehension theory, according to which older adults have greater difficulty in integrating semantic information with syntactic processing due to the lack of sufficient cognitive resources.

## Introduction

In sentence processing, the human brain makes use of both semantic and syntactic information to arrive at the correct representation of the sentence’s meaning (Traxler, 2011; Traxler, 2014). The ability to effectively integrate semantic information such as animacy with syntactic analysis is crucial for successful sentence comprehension. Animacy is an important issue that has received considerable attention in the studies of semantic-syntactic interplay in sentence processing. Regarded as an inherent property of nouns and a universal concept, animacy has often been examined in relation to syntactic information or other semantic concepts in languages and has been found to play an essential role in constructing grammatical relations (subject or object) and assigning thematic roles (agent or patient). Many studies have investigated the use of animacy cues in sentence processing among younger adults (e.g., Fedorenko & Gibson, 2008; Philipp et al., 2008; Traxler et al., 2005; Wu, Kaiser & Andersen, 2012), but little research has been conducted to explore sentence processing in older adults. Whether, and if so, how older adults use animacy cues in sentence processing in a way different from younger adults is not entirely clear. The present study intended to investigate the age differences in the use of animacy cues in the processing of Mandarin sentences.

In the domain of sentence processing, relative clauses have frequently been used to examine how individuals in different age groups processed sentences differently. Most prior studies have found that the comprehension of relative clauses became less accurate and efficient in normally ageing adults (e.g., Caplan et al., 2011; Kemtes & Kemper, 1997; Stinemorrow, Noh & Shake, 2010; Stinemorrow, Ryan & Leonard, 2000; Waters & Caplan, 2005). As prior research has found that the animacy of the sentential subjects or objects had a significant effect on the comprehension of relative clauses (Traxler, Morris & Seely, 2002; Traxler et al., 2005), relative clauses were used as the target structures in this study to explore how the effect of animacy on relative clause processing differed between younger and older adults.

Relative clauses can be divided into subject-extracted relative clauses (SRCs) and object-extracted relative clauses (ORCs) according to the extraction site. Studies from younger adults have reached a consensus that ORCs, such as (2), are more complex and more difficult to process than SRCs, such as (1). In sentence (1), *driver* is the agent of *hit* and *passenger* is the patient of *hit*. Sentence (2) is different from sentence (1) in that *driver* is the patient of *hit* while *passenger* is the agent of *hit.*

(1). The driver who hit the passenger admitted the error. (SRC)

(2). The driver who the passenger hit admitted the error. (ORC)

As both *driver* and *passenger* are animate nouns, according to our world knowledge, both *driver* and *passenger* can serve as the agent or patient of *hit*. In this case, we rely mostly on syntactic information to comprehend the sentences because semantic information cannot provide us helpful cues to understand who hit whom.

The effect of animacy cues can be illustrated by comparing the ORCs (3, 6) with the SRCs (4, 5).

(3). The director that the movie pleased received applause. (ORC-animate)

(4). The director that watched the movie received applause. (SRC-animate)

(5). The movie that pleased the director received applause. (SRC-inanimate)

(6). The movie that the director watched received applause. (ORC-inanimate)

Unlike (1) and (2), the animacy of the nouns in sentences (3–6) is contrastive. The RC verbs in the four sentences were different, with *watch* as an agentive verb and *please* as a causative psych-verb. The two types of verbs have different animacy restrictions on the argument nouns. Agentive verbs such as *watch*, *buy* or *kill* prefer the subjects to be animate whereas causative psych-verbs such as *please*, *frighten* or *terrify* allow the subjects to be inanimate (Pesetsky, 1995). In the four sentences (3–6), both *director* and *movie* can satisfy the animacy requirements of the verbs and serve as the legitimate subjects of the relative clauses. However, previous studies have suggested that there is a strong tendency in sentence processing to assign agent roles to animate nouns (*director*) and patient roles to inanimate nouns (*movie*) (Bock & Warren, 1985; Folli & Harley, 2008; Van Valin & LaPolla, 1997; Hale & Keyser, 2002). Animate nouns are in a better position to function as the subjects of sentences while inanimate nouns are more inclined to occupy the object positions (Bock & Warren, 1985; Folli & Harley, 2008; Givón & Whitaker, 1979). This tendency is so strong that it can override the animacy preference of the verbs. When the preferred animacy configuration (animate RC subject and inanimate RC object) is violated, as in (3) and (5), the sentences become more difficult to process.

Numerous studies have found that when nouns in RCs are both animate, ORCs are more difficult to process than SRCs (Andrews, Birney & Halford, 2006; Caplan & Waters, 1999; Caplan et al., 2011; King & Just, 1991; Traxler et al., 2005; Gordon, Hendrick & Levine, 2002; Andrews & Todd, 2008). In previous studies, the asymmetrical processing difficulty is usually attributed to the greater syntactic complexity or working memory demand of ORCs over SRCs (Frazier, 1985; Gibson, 2000; Carpenter, Miyake & Just, 1994; Gibson, 2000) or the violation of expectations (e.g., Hale, 2001; Levy, 2008; Levy et al., 2009). Many studies provided empirical evidence for both the memory-based account and the expectation-based account for the comparatively higher level of processing difficulty of ORCs over SRCs (e.g., Staub, 2010; Levy, Fedorenko & Gibson, 2013). Regarding the animacy effect, previous studies found that when the animacy cue is consistent with the syntactic analysis as in the case of (4) and (6), sentence processing becomes easier and contrarily, sentence processing is more difficult when animacy contradicts the syntactic analysis (King & Just, 1991; Traxler et al., 2005). In the example above, (4) and (6) should be easier to understand than (3) and (5) as they are consistent with the results of syntactic analysis. This difference in processing difficulty might be particularly prominent in syntactically complex sentences, such as ORCs (Ferreira, 2003).

The studies of animacy effects on RC processing (King & Just, 1991; Mak, Vonk & Schriefers, 2002; Mak, Vonk & Schriefers, 2006; Wu, Kaiser & Andersen, 2012) generally found that animacy influenced the parsing of RCs, which can be reflected in their modulation of the processing difficulty of SRCs and ORCs. Using a self-paced reading task and eye tracking, Mak, Vonk & Schriefers (2002) investigated the effect of animacy on relative clause processing and found that under the preferred animacy configurations (animate RC subject and inanimate RC object), the differences between SRCs and ORCs became insignificant. Thus the authors concluded that semantic information overrode the biases of syntactic analysis. Traxler, Morris & Seely (2002) found that when the sentence-initial subjects were animate, ORCs were more difficult to process than SRCs. However, when sentence-initial subjects were inanimate, there were no significant differences in processing difficulty between SRCs and ORCs. This study showed that inanimate head nouns reduced the processing difficulty of ORCs and consequently narrowed the gap between SRCs and ORCs. Studies of Dutch and Spanish relative clauses provided similar evidence showing that animacy has a crucial impact on sentence processing (Betancort, Carreiras & Sturt, 2009; Mak, Vonk & Schriefers, 2006).

All the studies described above focused on younger adults and few studies examined how older adults used animacy cues in relative clause processing. Given the significant decline in working memory, processing speed or inhibitory control among older adults (Altmann & Kemper, 2006), they might use animacy cues in a way different from younger adults. So far there has been only one study that examined the effect of animacy on relative clause processing by older and younger adults. Using a self-paced listening task, DeDe (2015) compared the performance of older and younger adults in the processing of English relative clauses by manipulating animacy configuration (animate, inanimate) and RC type (SRC, ORC). Results showed that both younger and older participants had longer listening times for the critical segments in ORCs than in SRCs regardless of the animacy conditions. However, the manipulation of animacy disrupted older adults more than younger adults. Compared with younger adults, older adults were more sensitive to animacy and more reliant on animacy to provide clues for sentence processing. The study concluded that older adults relied on animacy cues to make predictions so as to compensate for the age-related decline in sentence comprehension.

However, studies of other sentence structures offered evidence against this claim. Oh, Sung & Sim (2016) examined the differences between older and younger adults in animacy effects on the processing of Korean simple sentences with different word orders (SOV vs. OSV). Results found that animate nouns elicited larger N400 than inanimate nouns among younger adults in the object positions in SOV structures, but inanimate nouns elicited larger N400 than animate nouns in the sentence-initial object positions in OSV sentences. However, no significant animacy effect was found among older adults, but only a deferred N400 effect. This study showed that the effect of animacy on sentence processing was associated with the order of animacy. Violation of the “animate agent-inanimate patient” configuration would incur higher processing costs. Compared with younger adults, older adults processed animacy less efficiently. This is also supported by the studies of language production. Altmann & Kemper (2006) examined whether older and younger adults had similar preferences for animate nouns as sentential subjects and whether they relied on the order of argument activation to determine sentence structure in sentence production tasks. The results indicated that both older and younger adults tended to use animate nouns as sentential subjects. Critically, compared with younger adults, older adults were less sensitive to noun animacy and more sensitive to the order of activation. According to this study, older adults used semantic cues less efficiently in sentence production. However, Altmann & Kemper (2006) explored the role of animacy in language production and it is not clear whether their findings can be generalized to language comprehension.

The review of studies above shows that whether there is an age-related decline in the use of animacy cues in RC processing is still under debate. The present study intended to further clarify this issue by examining the effect of animacy on Mandarin relative clause processing among different age groups. Mandarin Chinese is a language typologically different from Indo-European languages and Mandarin relative clauses are uniquely head-final structures with the head nouns coming after the relative clauses. This structure makes it impossible for the head nouns to guide the parsing of relative clauses, as relative clauses are ahead of the head nouns. Additionally, as Mandarin has flexible word order and no overt morphological inflection, the influence of semantic factors such as animacy might be particularly strong due to the lack of other cues to guide sentence processing (Chen, 1984; Chu, 1998; Li & Thompson, 1981). Given these structural differences, the findings from Mandarin can provide additional evidence to cross-validate the findings from participants speaking English or other languages and allow us to view the effects of ageing on sentence processing from a cross-linguistic perspective.

### Theoretical predictions for age differences in animacy effects

To account for the animacy effect in relative clause processing, previous studies have proposed various theories with different focuses, from which we have identified two theories relevant to age differences in animacy effects on relative clause processing, namely the risky strategy hypothesis (DeDe, 2015) and the capacity constrained comprehension theory (Just & Carpenter, 1992; Just, Carpenter & Keller, 1996). The two theories view animacy from different perspectives and make contrastive predictions correspondingly.

#### The risky strategy hypothesis

The risky strategy hypothesis (DeDe, 2015) offered an experience-based account for language processing among older adults. DeDe (2015) proposed that there is a stronger reliance on probabilistic cues in sentence comprehension in older adults. Probabilistic cues refer to the statistical likelihoods about the most likely semantic or syntactic contexts in which certain words will occur (Traxler, Morris & Seely, 2002). For instance, some verbs in English usually appear in transitive constructions (*The policeman saw the thief*) whereas others typically occur in intransitive structures (*The girl danced in the garden*). These cues can be used to generate predictions about the upcoming words in the sentences, which may further influence online sentence processing (MacDonald, 1994). Animacy is a kind of probabilistic cue as the entity performing an action is more likely to be animate rather than inanimate (Traxler, Morris & Seely, 2002). Older adults have richer language experience and a greater accumulation of vocabulary knowledge than younger adults, which will give them an advantage in the use of probabilistic cues such as animacy cues in sentence processing. Older adults tend to take this advantage to make their performance comparable with younger adults. In online processing, older adults rely more on probabilistic cues to predict the upcoming words in the sentences, which is a very risky strategy because they will be more disrupted if their predictions are not correct (DeDe, 2015).

DeDe (2015) maintained that probabilistic cues related to the role of animate and inanimate nouns influence how difficult it was to process relative clauses. On this account, older adults should show increased processing disruptions when the animacy cues are inconsistent with the interpretation of the sentences. For example, in processing sentences such as (3), older adults relied on the probabilistic cues to predict that the animate noun *director* should be the agent of the relative clause verb. However, their prediction turned out to be incorrect as *director* actually served as the patient of the verb *pleased*, which resulted in greater difficulties for older adults. Thus, the effect of ageing would be stronger in (3) than in (6). DeDe (2015) compared the performance of older and younger adults in processing English relative clauses using a self-paced listening task and found that when the sentential subjects were animate in object relative clauses, a situation which violated the preferred animacy configuration, older adults encountered greater difficulties than younger adults.

The author concluded that older adults relied more on experience-based predictions than younger adults in sentence processing and were more sensitive to animacy constraints. According to this hypothesis, compared with younger adults, the influence of animacy configuration on the processing difficulty of relative clauses would be greater among older adults.

#### The capacity constrained comprehension theory

The capacity constrained comprehension theory (Just & Carpenter, 1992; Just, Carpenter & Keller, 1996), which offered a resource-based account for sentence processing, proposed that language comprehension is subject to the constraint of working memory resources. Syntactic processing and other non-syntactic processes such as semantic or pragmatic processing share a single pool of working memory resources. Subjects with high working memory capacity possess enough resources to keep both syntactic information and non-syntactic information simultaneously activated and thus to integrate them more efficiently than those with low working memory capacity. Limitations in working memory lead to greater boundaries between different processes and create stronger encapsulation and modularity of syntactic processing. To confirm this claim, Just & Carpenter (1992) used eye tracking to compare the use of animacy information in the processing of reduced and unreduced relative clauses between subjects with high working memory capacity and those with low working memory capacity. The study found that the subjects with high working memory capacity were more sensitive to animacy in the first time passing whereas those with low working memory capacity did not have sufficient resources to use animacy cues. Just & Carpenter (1992) pointed out that this theory could also be used to account for the age-related differences in sentence processing, particularly in the tasks which place a large demand on working memory, such as the comprehension of embedded clauses. According to the capacity constrained comprehension theory, older adults lack sufficient resources to integrate animacy with syntactic processing, resulting in great difficulties in using animacy cues. Older adults are less sensitive to animacy cues in online sentence processing due to their reduced working memory capacity.

The two theories summarized above make contrastive predictions regarding the age differences in animacy effects on sentence processing. The present study aimed to empirically test the predictive power of the two theories by examining the processing of relative clauses in Mandarin, a typologically unique language which provides an ideal testing ground for distinguishing the adequacy of the two theories.

#### The present study

This study investigated the differences between older and younger adults in integrating animacy cues with syntactic analysis in the processing of Mandarin RCs. Specifically, we examined how older and younger adults differentially used animacy cues in processing SRCs and ORCs. According to Gibson’s (1998) Dependency Locality Theory, the processing difficulty or complexity of a syntactic structure is determined by the computational resources that the structure requires to process. The Dependency Locality Theory proposed that there are two kinds of processing costs: integration cost and storage cost. The integration cost is a function of the linear distance between the gap and the filler or the number of intervening discourse referents between the two. The storage cost is determined by the number of upcoming heads predicted for a complete syntactic dependency relationship. In the example (7–8) below, the head noun *nanhai* (‘boy’) is the filler. According to the generative grammar, relative clauses are formed through syntactic movement. The noun *nanhai* is moved from its original RC subject or object position to the sentential subject position, leaving behind it a trace or gap. The filler *nanhai* needs to be linked with the gap to arrive at the correct interpretation of the sentence. As the linear distance between the gap and the filler is longer in Mandarin SRCs (7) than in ORCs (8), and SRCs require more predicted heads than ORCs, SRCs have higher integration costs and storage costs than ORCs (Hsiao & Gibson, 2003). Thus SRCs were regarded as syntactically more complex, whereas ORCs were considered less complex in the present study.

(7). zhuigan xiaogou de  nanhai shuaidaole (SRC)

chase dog de boy fall down-le

‘The boy who chased the dog fell down.’

(8). xiaogou zhuigan de  nanhai shuaidaole (ORC)

dog chase de boy fall down-le.

‘The boy who the dog chased fell down.’

This study intended to find out whether the processing difficulty of RCs was modulated by the use of animacy cues among older and younger adults. If older adults can use animacy cues more effectively than younger adults, as predicted by the risky strategy hypothesis, the asymmetry in processing difficulty between SRCs with the preferred animacy configuration (inanimate RC-internal noun and animate main clause subject) and ORCs with the non-preferred animacy configuration (inanimate RC-internal noun and animate main clause subject) would be reduced, eliminated or even reversed to a greater extent in older adults. In other words, older adults would show a more dramatic change in processing difficulty of RCs compared with younger adults. Contrarily, if there is a decline in the use of animacy cues among older adults, as predicted by the capacity constrained comprehension theory, the processing difficulty would be less modulated by animacy in the older group and a relatively more dramatic change in processing difficulty would be witnessed in the younger group. In the current research, an experiment was designed to test the two completing claims.

## Method

### Design

The experiment adopted a 2 (animacy: animate, inanimate) ×2 (RC type: SRC, ORC) ×2 (age: old, young) design. Age is a between-subjects variable. Animacy and RC type are within-subjects variables. The accuracy of comprehension and reading times are the dependent variables.

### Participants

62 participants were recruited to take part in this study, including 31 older adults and 31 younger adults. The data from one younger participant were not saved due to a computer error. The older participants were aged between 60 and 80 years (Mean = 66.42, SD = 3.81) and the younger group were between 18 and 33 years old (Mean = 19.66, SD = 0.92). All participants spoke Mandarin Chinese as their native language and all were right-handed with normal vision or correct-to-normal vision. The two groups were well matched in gender ratio, (Chi-square Test, *χ*^2^ = .061, *df*  = 1,  *p* = .806) and years of education, *t*(59) = 1.51, *p* = .137. The demographic information was summarized in Table 1. Participants gave verbal consent to the experiment and received 30 RMB for their participation after the experiment. The experiment was approved by the Ethics Committee of the Beijing Foreign Studies University in China.

**Table 1:** Demographic information.

**Group**	**N**	**Sex**	**Age**	**Education**	**VWM**
young	30	female 24	19.66(0.92)	13.93(1.13)	4.60(0.97)
old	31	female 24	66.42(3.81)	13.06(3.08)	3.94(0.85)
Sig.	–	.806	.000^***^	.137	.006^**^

**Notes.**

VWM, Verbal working memory.

** *p* < .01.

*** *p* < .001.

### Neuropsychological tests

Before the experiment, all older adults were screened for cognitive health with the Chinese version of the Mini-Mental State Examination (CMMSE; Katzman et al., 1988), which was a relatively direct translation of Folstein, Folstein & McHugh (1975)’s MMSE. Katzman et al. (1988) used CMMSE for a dementia screening survey among a sample of 5,055 Chinese older adults and found that it has worked as an effective survey tool in the majority of respondents despite the cultural differences. The test was administered in face-to-face interviews with the participants. All participants were found to be cognitively healthy (CMMSE ≥ 26).

Participants’ verbal working memory capacity was assessed with Daneman & Carpenter’s (1980) experimental paradigm. We followed the same procedures as Daneman & Carpenter’s (1980) reading span task. Participants were required to read a group of sentences and recall the final words of the sentences at the end of each group. We used the Mandarin sentences from Cui & Chen’s (1996) study. All sentences were compound sentences containing 16 to 18 two-character Mandarin words. The final words of the sentences were concrete nouns, as abstract words might affect the difficulty to recall (Mackey et al., 2010). The participants completed the working memory test individually in a quiet room. Before the task, instructions were given in Mandarin on how to perform the test. Participants were told that their performance on the recall questions and the comprehension questions was equally important. The number of sentences in each group ranged from two to seven. All the groups were presented to participants in an ascending order, which means that the two-sentence group was presented first, followed by three-, four-, and five-sentence groups. Two groups were used for practice before the test started, with each group containing two sentences. The total score was the maximum number of sentences in the group which they can read while correctly recalling the final words. The results indicated that young adults’ verbal working memory span was significantly larger than that of older adults, t(59) = 2.84 , *p* < .05.

### Materials

The experimental stimuli were Mandarin sentences with a typical subject-verb-object structure and embedded relative clauses. The two factors manipulated in this experiment were the type of RC (SRC or ORC) and animacy configuration (animate main clause subject, inanimate main clause subject), which resulted in four conditions as exemplified in Table 2. Specifically, the four experimental conditions were subject relative clauses with animate main clause subjects (SRC animate), subject relative clauses with inanimate main clause subjects (SRC inanimate), object relative clauses with animate main clause subjects (ORC animate) and object relative clauses with inanimate main clause subjects (ORC inanimate). All sentences used contrastive animacy configuration, which means that the nouns within the relative clauses were inanimate when the main clause subjects were animate, and the nouns within the relative clauses were animate when the main clause subjects were inanimate. By using contrastive animacy, we were able to compare the preferred animacy configuration (animate RC subject, inanimate RC object) with the non-preferred one (inanimate RC subject, animate RC object). This design was to increase the animacy differences between the different experimental conditions so as to maximize experimental variance. In this design, when two types of relative clauses had the same head, as *jizhe* in (7) and (8), or *xinwen* in (9) and (10), they also had the same nouns within the relative clause regions. All the four conditions had the same main clause verb (*yinqi*), main clause object (*minzhongdezhuyi*) and RC-internal verb (*baoguang*). This design allowed for the minimization of lexical differences across the four conditions. The experimental stimuli were adapted from the sentences used by He & Chen (2013) and Wu, Kaiser & Andersen (2012). Unlike many previous studies which have manipulated the RC verbs to allow the subjects and objects to be reversible (e.g., Mak, Vonk & Schriefers, 2006; DeDe, 2015), we chose to use the same RC verbs across the four experimental conditions in order to exclude any confound of word frequency, word meaning, and number of strokes per character. This design allowed us to compare different experimental conditions while controlling for potential confounders such as the lexical semantic features of the RC verbs.

**Table 2:** Sample stimuli.

**Condition**	**Stimuli**
SRC animate	a. baoguang xinwen de jizhe yinqile minzhongdezhuyi
	b. expose news DE reporter attract people’s attention
	c. The reporter who exposed the news attracted people’s attention.
SRC inanimate	a. baoguang jizhe de xinwen yinqile minzhongdezhuyi
	b. expose reporter DE news attract people’s attention
	c. The news which exposed the reporter attracted people’s attention.
ORC animate	a. xinwen baoguang de jizhe yinqile minzhongdezhuyi
	b. news expose DE reporter attract people’s attention
	c. The reporter who the news exposed attracted people’s attention.
ORC inanimate	a. jizhe baoguang de xinwen yinqile minzhongdezhuyi
	b. reporter expose DE news attract people’s attention
	c. The news which the reporter exposed attracted people’s attention.

**Notes.**

SRC animate: subject relative clause with animate main clause subject.

SRC inanimate: subject relative clause with inanimate main clause subject.

ORC animate: object relative clause with animate main clause subject.

ORC inanimate: object relative clause with inanimate main clause subject.

120 experimental sentences were used, including 30 sentences in each condition. Sentences were of equal length and were all segmented into six regions. As Table 2 shows, Segment 1 was the RC verb in subject relative clauses or RC subject in object relative clauses and Segment 2 was the RC object in subject relative clauses or RC verb in object relative clauses. Segment 3 was the RC marker *de* and Segment 4 was the main clause subject. In Segment 5, the grammatical aspect marker *le* was combined with the main verb as a single region. The last segment was the main clause object. 120 fillers which were of various length and structures were also implemented in the experiment. All 240 sentences were divided into four blocks with 60 sentences in each block. There were short breaks between two blocks. The sentences were all pseudo-randomized before being presented to the participants. See Table 2 for the sample sentences (a), the glossed sentences (b) and the English equivalents (c).

All experimental sentences and filler sentences were followed by comprehension questions. The questions asked about different parts of the sentences in order to encourage the participants to focus equally on all parts of the sentences. Half of the questions were designed to ask about the relative clause regions and the other half were about the main clause regions. For example, for the sample sentences in Table 2, the comprehension question could be either *jizhe baoguangle xinwen ma?* (‘Did the reporter expose the news?’), or *xinwen yinqile minzhongdezhuyi ma?* (‘Did the news attract people’s attention?’). Half of the questions had “yes” answers, and half had “no” answers. The expected yes/no answers were counterbalanced across conditions.

To ensure that there was no difference in the semantic plausibility of the sentences between the four conditions, we designed a sentence plausibility rating survey and invited 28 adults (14 old, 14 young) to rate the plausibility of the experimental sentences according to a five-point scale with scores ranging from “1” (the least natural) to “5” (the most natural). Those who participated in the survey did not take part in the experiment. The results indicated that there was no significant difference in plausibility ratings between the four types of relative clauses (SRC-animate: Mean = 3.20, ORC-inanimate: Mean = 3.34, SRC-inanimate: Mean = 3.17, ORC-animate: Mean = 3.25, *F* (1, 27) = 1.38, *p* = .246).

### Procedure

The experiment was designed with E-Prime. The experiment consisted of a practice session and an experiment session. Participants were required to perform a self-paced reading task in which they read sentences one word at a time and pressed the spacebar to indicate that they finished reading each segment, at which point the next word appeared on the screen and the previous word disappeared. They were required to read at a natural speed. At the end of each sentence, participants were asked to answer a comprehension question regarding the sentence they have just read and respond to the question as quickly as possible with a press of the button (“1” for YES and “0” for NO). Then a fixation “+” appeared at the center of the screen signaling the beginning of a new sentence. Participants completed eight practice trials before proceeding to the formal experiment. They were allowed to practice repeatedly until they were familiar with the operations. The computers recorded how long it took the participants to respond to each segment and whether their responses to the comprehension questions were accurate.

### Data analysis

In this study, mixed-effects modeling was applied to assess the effects of experimental factors, using the lme4 package (Bates et al., 2014) in R (R Development Core Team, 2014). Linear models were used for reading times and a logistic model was used for the accuracy data. Predictor variables included the within-subjects factors animacy (animate, inanimate) and RC type (SRC, ORC), between-subjects factor age (old, young) and all interactions. Random intercepts were included for subjects and items. The p-values were estimated with Satterthwaite’s approximation. For post-hoc pairwise comparisons reported in this study, Tukey’s HSD tests were implemented using lsmeans package (Lenth, 2015).

Statistical analysis was performed for the relative clause region, the relative clause marker *de*, the head noun and the main verb. The critical regions of interests for reading time analysis included the relative clause segments, the RC marker *de* and main clause subjects. Reading times for main clause verbs were also analyzed to explore the possible spillover effects from the preceding regions. As the semantic and syntactic features for Segment 1 (RC noun/verb) and Segment 2 (RC verb/noun) were different across the four conditions, we collapsed the two segments to analyze them as a single entity, as Hsiao & Gibson (2003), He & Chen (2013), and Vasishth et al. (2013) did in their studies. Reading times that were beyond 2 standard deviations of the mean for each participant in each condition were treated as outliers and removed from the subsequent data analysis.

## Results

### Accuracy

**Figure 1:**
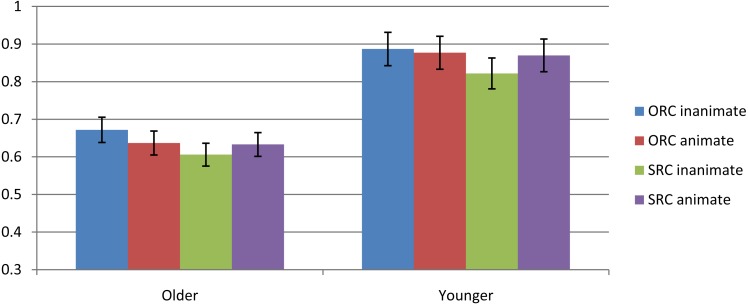
Mean accuracy by age group.

The mean accuracy of sentence comprehension by age group is graphically summarized in Fig. 1. The results of the regression models are summarized in Table 3. The overall mean accuracy for older adults was 63.7 percent and the mean accuracy for younger adults was 86.4 percent. Older adults showed much lower accuracy than younger adults. However, the fact that the accuracy was over 50 percent suggested that the older adults’ performance was not at random. Once the accuracy was computed for each participant in each condition, a *t*-test was conducted to determine if the participants performed reliably above than the chance level. The results indicated that both older adults and younger adults performed significantly above chance level old: *t* = 6.41, *p* < .05; young: *t* = 27.89, *p* < .05.

**Table 3:** The output of logistic mixed effects modeling for the accuracy data.

	**Estimate**	**St. Error**	***z*-value**	Pr (>—*z*—)
(Intercept)	0.756	0.117	6.421	<.0001^***^
Age group	1.405	0.189	7.443	<.0001^***^
Animacy	−0.161	0.112	−1.448	0.1475
RC type	−0.297	0.111	−2.690	0.0071^**^
Age group^*^ Animacy	0.070	0.199	0.355	0.7227
Animacy^*^ RC type	0.281	0.156	1.804	0.0712
Age group^*^ RC type	−0.249	0.190	−1.309	0.1904
Age group^*^ Animacy^*^ RC type	0.198	0.272	0.726	0.4681

**Notes.**

* Significance codes: 0.05.

** Significance codes: 0.01.

*** Significance codes: 0.001.

The results of logistic mixed-effects regression modeling indicated that there was a significant effect of age, *β* = 1.40, *SE* = 0.19, *z* = 7.44, *p* < .05, a significant effect of RC type, *β* =  − 0.30, *SE* = 0.11, *z* =  − 2.70, *p* < .05, and a marginally significant interaction between animacy and RC type, *β* = 0.28, *SE* = 0.15, *z* = 1.80, *p* = .071. There was no significant effect of animacy or three-way interaction effect between age, RC type and animacy (*p* s > .05). In older adults, the accuracy for SRCs was significantly lower than that for ORCs when the main clause subjects are inanimate (*p* < .05). When the main clause subjects were animate, there was no significant effect of RC type (*p* = .979). In younger adults, when the main clause subjects are inanimate, the accuracy for SRCs was significantly lower than that for ORCs (*p* < .05), indicating that SRCs were more difficult to comprehend than ORCs. When the main clause subjects were animate, there was no significant difference in accuracy between the two types of RCs (*p* = .706). The results indicated that in both younger and older adults, the asymmetrical processing difficulty of SRCs and ORCs was modulated by animacy configurations. Thus both groups made use of the animacy cues in sentence comprehension and there was no age-related decline in the accuracy of responses to comprehension questions.

### Reading time

Reading times beyond two standard deviations of the mean for each participant in each condition were excluded, resulting in 5.09% of the data removed. The average reading times for each segment are presented in Table 4.

**Table 4:** Mean reading times (in ms).

**Group**	**Condition**	**RCverb/noun**	RC noun/verb	**DE**	**Mainsubject**	**Mainverb**	**Mainobject**
		mean(sd)	mean(sd)	mean(sd)	mean(sd)	mean(sd)	mean(sd)
old	ORC-inanimate	648 (287)	521(223)	502(263)	551(336)	621(437)	1,180(881)
	ORC-animate	673(317)	523(231)	497(271)	558(404)	630(523)	1,243(956)
	SRC-inanimate	719(361)	517(223)	490(267)	565(397)	633(510)	1,219(974)
	SRC-animate	664(305)	512(203)	486(300)	567(503)	617(431)	1,246(975)
young	ORC-inanimate	333(107)	357(139)	299(192)	301(181)	365(370)	467(304)
	ORC-animate	332(107)	388(187)	371(216)	427(346)	483(514)	585(343)
	SRC-inanimate	330(111)	451(314)	478(348)	647(611)	612(413)	777(468)
	SRC-animate	332(112)	447(251)	359(183)	376(224)	435(297)	533(326)

The results of regression modeling for reading times at relative clause segments are summarized in Table 5. In the relative clause segment, there was a significant effect of age, *β* =  − 0.45, *SE* = 0.09, *t* =  − 4.92, *p* < .05, a significant effect of RC type, *β* = 0.04, *SE* = 0.02, *t* = 1.65, *p* < .05, a significant interaction effect between age and RC type, *β* = 0.08, *SE* = 0.03, *t* = 3.25, *p* < .05, and a marginally significant interaction effect between animacy and RC type, *β* =  − 0.05, *SE* = 0.03, *t* =  − 1.45, *p* = .083. The effect of animacy and the three-way interaction between age, type and animacy were not significant (*p* s >.05). Pairwise comparison found that older adults had significantly longer reading times than younger adults (*p* < .05). In the younger adults, when the main clause subjects are inanimate, SRCs were processed more slowly than ORCs (*p* < .05). When the main clause subjects are animate, there was no significant difference between SRCs and ORCs (*p* = .950). In the older adults, SRCs were processed more slowly than ORCs regardless of the animacy conditions (*p* s <.05).

**Table 5:** The output of linear mixed effects model for the reading times at RC regions.

	**Estimate**	**St. Error**	***t*-value**	Pr (>—*z*—)
Age group	−0.452	0.091	−4.924	<.0001^***^
Animacy	0.030	0.026	1.176	0.5878
RC type	0.041	0.025	1.654	0.0008^***^
Age group^*^ Animacy	0.005	0.024	0.214	0.8385
Animacy^*^ RC type	−0.048	0.034	−1.445	0.0834
Age group^*^ RC type	0.079	0.025	3.253	<.0001^***^
Age group^*^ Animacy^*^ RC type	−0.003	0.035	−0.099	0.9208

**Notes.**

* Significance codes: 0.05.

** Significance codes: 0.01.

*** Significance codes: 0.001.

The results of RT analysis for the relative clause marker *de* are summarized in Table 6. In these segments, we found a significant effect of age, *β* = −0.49, *SE* = 0.08, *t* =  − 5.84, *p* < .05, a significant interaction effect between RC type and animacy, *β* = 0.02, *SE* = 0.04, *t* = 0.43, *p* < .05, a significant interaction effect between RC type and age, *β* = 0.39, *SE* = 0.03, *t* = 14.96, *p* < .05, and a significant interaction effect between age, animacy and sentence type, *β* =  − 0.39, *SE* = 0.04, *t* =  − 10.39, *p* < .05. The effect of animacy and the interaction between age and animacy were not significant (*p* s > .05). Pairwise comparison revealed that in the older group, there was no significant difference between SRCs and ORCs in both animacy conditions. In the younger group, the reading times for SRCs were significantly longer than those for ORCs when the main clause subjects are inanimate (*p* < .05), but there was no significant difference between the two types of relative clauses when the main clause subjects are animate (*p* = .913).

**Table 6:** The output of linear mixed effects model for the reading times at RC Marker *de*.

	Estimate	St. Error	*t*-value	Pr ( >|*z*|)
Age group	−0.485	0.083	−5.843	<.0001^***^
Animacy	−0.016	0.027	−0.589	0.8382
RC type	−0.031	0.026	−1.205	<.0001^***^
Age group^*^ Animacy	0.205	0.027	7.703	0.5824
Animacy^*^ RC type	0.015	0.035	0.429	<.0001^***^
Age group^*^ RC type	0.395	0.026	14.963	<.0001^***^
Age group^*^ Animacy^*^ RC type	−0.389	0.037	−10.396	<.0001^***^

**Notes.**

* Significance codes: 0.05.

** Significance codes: 0.01.

*** Significance codes: 0.001.

Table 7 summarized the results of RT analysis for main clause subjects. In this segment, there was a significant effect of age, *β* =  − 0.55, *SE* = 0.09, *t* =  − 5.99, *p* < .05, a significant effect of RC type, *β* = 0.01, *SE* = 0.02, *t* = 0.45, *p* < .05, a significant interaction effect of RC type and animacy, *β* =  − 0.01, *SE* = 0.03, *t* =  − 0.44, *p* < .05, a significant interaction effect between RC type and age, *β* = 0.56, *SE* = 0.03, *t* = 17.41, *p* < .05, and a significant interaction effect between age, animacy and sentence type, *β* =  − 0.63, *SE* = 0.05, *t* =  − 13.78, *p* < .05. The effect of animacy and the interaction between age and animacy were not significant (*p* s >.05). Pairwise comparison showed that the younger adults had longer reading times in SRCs than in ORCs in the inanimate condition (*p* < .05). In the animate condition, there was no significant difference in reading times between SRCs and ORCs (*p* = .131). In the older group, no significant difference was found between SRCs and ORCs in either animacy condition (*p* s >.05).

**Table 7:** The output of linear mixed effects model for the reading times at main clause subject.

	**Estimate**	**St. Error**	*t*-value	Pr ( >|*z*|)
Age group	−0.551	0.092	−5.989	0.0023^**^
Animacy	−0.004	0.027	−0.148	0.1413
RC type	0.010	0.022	0.448	<.0001^***^
Age group^*^ Animacy	0.284	0.033	8.715	0.1699
Animacy^*^ RC type	−0.014	0.032	−0.442	<.0001^***^
Age group^*^ RC type	0.561	0.032	17.408	<.0001^***^
Age group^*^ Animacy^*^ RC type	−0.631	0.046	−13.780	<.0001^***^

**Notes.**

* Significance codes: 0.05.

** Significance codes: 0.01.

*** Significance codes: 0.001.

As Table 8 shows, in the main verb segment, there was a significant effect of age, *β* =  − 0.54, *SE* = 0.09, *t* =  − 5.95, *p* < .05, a significant interaction effect between RC type and animacy, *β* =  − 0.01, *SE* = 0.03, *t* =  − 0.32, *p* < .05, a significant interaction effect between RC type and age, *β* = 0.49, *SE* = 0.03, *t* = 15.19, *p* < .05, and a significant interaction effect between age, animacy and sentence type, *β* =  − 0.53, *SE* = 0.04, *t* =  − 11.86, *p* < .05. The effect of animacy and the interaction between age and animacy were not significant (*p* s > .05). SRCs were processed more slowly than ORCs in older adults, but the difference was not statistically significant (*p* > .05). In the younger group, there was a significant effect of sentence type in the inanimate condition (*p* < .05), but the effect was not significant in the animate condition (*p* = .168).

**Table 8:** The output of linear mixed effects model for the reading times at main clause verbs.

	**Estimate**	**St. Error**	*t*-value	Pr ( >|*z*|)
Age group	−0.535	0.089	−5.949	0.0013^**^
Animacy	0.002	0.023	0.077	0.5597
RC type	0.004	0.022	0.167	<.0001^***^
Age group^*^ Animacy	0.263	0.032	8.142	0.7678
Animacy^*^ RC type	−0.010	0.032	−0.320	<.0001^***^
Age group^*^ RC type	0.486	0.032	15.191	<.0001^***^
Age group^*^ Animacy^*^ RC type	−0.530	0.045	−11.857	<.0001^***^

**Notes.**

* Significance codes: 0.05.

** Significance codes: 0.01.

*** Significance codes: 0.001.

## Discussion

Most previous studies focused on how younger adults used animacy in relative clause processing, it is still far from clear how the ability to use animacy information changes with advancing age. The present study investigated the age-related changes in animacy effects on the comprehension of Mandarin relative clauses. The results indicate that younger adults were generally more sensitive to animacy information in RC processing. Specifically, in the younger group, animacy was found to modulate the processing difficulty of SRCs and ORCs in all segments in online processing. SRCs were more difficult to process than ORCs when the main clause subjects were inanimate, but there was no significant difference in processing difficult between SRCs and ORCs when the main clause subjects were animate. This finding was supported by He & Chen’s (2013) study which also found that young adults could use animacy cues in Mandarin RC processing. In the older group, no significant effect of animacy or interaction between animacy and RC type was found in any region in online processing. In the relative clause segments, SRCs were more difficult to process than ORCs regardless of the animacy conditions, which suggests that older adults relied on syntactic cues rather than animacy cues. No significant difference between SRCs and ORCs was found in the RC marker *de*, main clause subjects and verbs, indicating that neither animacy cues nor syntactic cues were used effectively in these segments. This is not exactly the same as our initial expectation as we expected to find an effect of RC type even if the animacy effect was missing. However, the lack of a significant animacy effect and interaction effects between animacy and any other variable can still provide strong evidence showing that older adults cannot use animacy cues as effectively as younger adults in online processing.

The ability to identify, recognize or process lexical semantic features such as animacy could decline in the aging population. The finding is consistent with the claim that older adults are less sensitive to the constraints of animacy compared with younger adults (Federmeier & Kutas, 2005; Traxler et al., 2005). The decline in sentence processing in older adults has been reported in several other studies which also found a similar decline in older adults’ sensitivity to animacy information in the processing of passive sentences (Oh, Sung & Sim, 2016) and simple sentences (Altmann & Kemper, 2006). This indicates that the age-related decline in animacy effects may not be specific to Mandarin relative clause processing. Rather it could be a universal phenomenon which can be found in the comprehension of other sentence structures and other languages as well.

However, the finding contradicts DeDe’s (2015) study which examined the animacy effect on the processing of English relative clauses by older and younger adults and discovered that compared with younger adults, older adults were more sensitive to animacy and tended to adopt more risky strategies to predict the upcoming words. There are several candidate explanations for the differences between DeDe’s (2015) study and our study. First, the divergent results might be attributed to the differences in participants’ educational experience. The older participants in DeDe’s (2015) study had more years of education and richer vocabulary knowledge than the younger participants while in our study, the older adults had slightly lower level of education than the younger adults, although the difference did not reach statistically significant level. The advantage in educational experience and vocabulary knowledge might enable the older adults in DeDe’s (2015) study to make better use of semantic cues in sentence processing. However, the older adults in our study may not have such advantage over the younger adults. Another possible reason is related to the individual differences in participants’ working memory. In the present study, older adults had lower working memory capacity than younger adults and consequently the reduced working memory could constrain their effective use of animacy cues. Older adults may not have sufficient cognitive resources to exercise the predictive processing strategies outlined in DeDe’s (2015) study. DeDe (2015) mainly emphasized how older adults made predictions based on their language experience and therefore did not take working memory into consideration. Working memory was neither measured nor analyzed in her study. If older and younger adults did not differ significantly in working memory capacity, older adults who had richer language experience might outperform younger adults in the use of animacy information in relative clause processing. This study, combined with DeDe’s (2015) study, seemed to indicate that older adults can rely on their language experience to integrate animacy into sentence processing, but this can only be done when they have sufficient cognitive resources available. Although semantic processing might be computationally less demanding, the integration of semantic information with syntactic analysis could consume working memory resources and as a result, the use of animacy may be constrained by the amount of working memory resources available.

Another finding of our study is that although there was an age-related decline in animacy effects on the word-by-word reading times, the older adults were able to use animacy cues just like the younger adults in offline sentence comprehension as reflected in the accuracy scores on questions probing comprehension of sentences. Although there was an effect of age on comprehension accuracy, such age effect did not interact with the effect of animacy or RC type, which suggested that there was no age-related difference in these effects. From the offline results, we found a significant interaction between animacy and RC type in both younger and older adults, indicating that both age groups were able to use animacy cues and older adults were not inferior to younger adults in the use of animacy cues. This suggested that both older and younger adults could ultimately take animacy into account, although only the younger adults could take it into account at the initial online processing stage. The ability to utilize semantic cues is less vulnerable to ageing in offline sentence comprehension. This finding was consistent with DeDe (2015)’s study. The results could be explained by the capacity constrained comprehension theory, according to which, there is little competition between different cognitive processes for the limited cognitive resources in offline processing (Just & Carpenter, 1992) and the demand for resources might not exceed the cognitive resources available for older adults. Consequently they have relatively sufficient cognitive resources to integrate animacy with syntactic processing. The finding, together with the results about online reading times, revealed the different time courses older adults and younger adults may take in using semantic cues in sentence processing. Older adults integrated all semantic cues from what they read to arrive at the best interpretations they could possibly achieve at the later stage of sentence comprehension, whereas younger adults could use animacy cues at the very early stage of sentence processing. Older adults might temporarily postpone the use of animacy cues due to insufficient cognitive resources available, which could be regarded as a language processing strategy to compensate for their working memory decline. The findings provided evidence showing older adults did not lose the competence to use animacy cues in sentence processing. Rather they merely suffered from a reduced efficiency to integrate animacy information with syntactic analysis in online processing.

When one considers the two theoretical accounts described earlier in this paper, it seems that the findings reported here were most consistent with the capacity constrained comprehension theory. The predictions of DeDe’s (2015) risky strategy hypothesis cannot fit straightforwardly with our results. On the whole, we found younger adults were better able to utilize animacy cues in relative clause processing than older adults, contrary to what is predicted by the risky strategy hypothesis. When applying DeDe’s (2014) risky strategy hypothesis to relative clause processing, we assumed that online parsing is influenced by language experience and that animacy cues guided parsing during reanalysis. As older adults have richer language experience and a better command of vocabulary knowledge, they rely more on animacy cues to make predictions in relative clause processing (DeDe, 2015). The findings from our study were contradictory to the prediction in that we found that it is younger adults, rather than older adults who had stronger reliance on animacy cues. It should be noticed that this theory cannot fully account for the findings from DeDe’s (2015) empirical study either. According to the predictions of this theory, older adults are more sensitive to animacy and consequently, stronger animacy effects should be identified in the processing of both ORCs and SRCs regardless of their syntactic complexity. However, DeDe (2015) found animacy effects among older adults only in the syntactically more complex ORCs while the processing of SRCs did not seem to display significant animacy effects. The risky strategy hypothesis alone cannot explain the asymmetrical animacy effects. As a matter of fact, we have not been able to find evidence from previous studies which can perfectly fit the prediction of this theory. The possible reason is that this theory is purely semantic-driven, ignoring the complex interplay between syntactic processing and semantic processing. However, in reality, relative clause processing is a complicated process with multiple factors such as syntactic, semantic and pragmatic factors interacting and integrated with each other and the picture becomes even more complex when the age factor is taken into consideration. Therefore, this theory has certain limitations when used to account for the age differences in the effect of animacy. This suggests that a purely semantic-driven account may not be sufficient.

The findings from the present study generally supported the capacity constrained comprehension theory which intended to account for sentence processing from the perspective of interaction between syntax and semantics. According to this theory, individuals with low working memory capacity cannot effectively integrate semantic information such as animacy into online sentence processing (Just & Carpenter, 1992; Just, Carpenter & Keller, 1996), resulting in a more modular processing pattern. The findings from our study can be easily explained in terms of a capacity difference between older and younger adults, such that only the younger adults had the capacity to take the animacy information into account in online processing. Due to working memory decline, older adults encountered greater difficulties in keeping both semantic and syntactic information activated in online sentence processing and consequently, their ability to integrate semantic information with syntactic analysis was largely compromised. As there was little competition between syntactic and semantic processing in the offline processes, their performance remained relatively unaffected. The capacity constrained comprehension theory can offer an adequate explanation for the age differences in the use of semantic cues in sentence processing.

## Conclusions

The current research examined the age-related differences in the effect of animacy on Mandarin sentence processing using a self-paced reading paradigm. The study suggests that younger adults were more sensitive to the animacy constraints in online sentence processing, which confirms the predictions of the capacity constrained comprehension theory. The findings provide evidence showing the use of animacy cues becomes less efficient in the aging population. Given that the semantic cues play a more important role than syntactic cues in sentence processing among Chinese-speaking subjects (Chen, 2006), the fact that the use of animacy information declines with advancing age might imply that Chinese-speaking older adults have greater difficulties with sentence comprehension compared with the adults speaking other languages. We hope future research could continue to shed light on the age-related decline in sentence comprehension among Chinese-speaking older adults.

As the current study only presented the behavioral evidence regarding the age-related animacy effects on Mandarin RC processing, further studies are needed to examine the neural mechanisms to gain a deeper understanding of the animacy effect on sentence processing. Moreover, we manipulated animacy information with only human and inanimate nouns. Considering the hierarchical nature of animacy, it might be necessary to extend the present study to other types of animacy information such as abstract concept or animal for future investigation. Finally, this study failed to include as many older subjects in higher education as younger subjects due to difficulties in recruitment. It is possible that the use of animacy cues might increase for the older adults with a higher level of education. Therefore, further research might examine the performance of the older adults with richer educational experience to better elucidate the relationships between aging and the use of lexico-semantic cues in Mandarin sentence processing.

##  Supplemental Information

10.7717/peerj.6437/supp-1 Supplemental Information 1 Performance of relative clause processing by younger and older adults

## Additional Information and Declarations

### Data Availability

The following information was supplied regarding data availability: The raw measurements are available in the Supplemental File.
